# Effectiveness of personalized rehabilitation in adults suffering from persistent concussion symptoms as compared to usual care: a randomized control trial protocol

**DOI:** 10.1186/s12883-024-03700-5

**Published:** 2024-07-10

**Authors:** Nicholas Moser, Milos R. Popovic, Sukhvinder Kalsi-Ryan

**Affiliations:** 1 KITE Research Institute-University Health Network, Toronto, ON Canada; 2 Temerty Faculty of Medicine, Institute of Medical Science, University of Toronto, Toronto, Nicholas Canada; 3 Institute of Biomedical Engineering, University of Toronto, Toronto, ON Canada; 4 Rehabilitation Sciences Institute, University of Toronto, Toronto, ON Canada

**Keywords:** Post-concussion syndrome, Concussion, Rehabilitation, Exercise, Headache

## Abstract

**Background:**

Symptoms reported by patients who sustain a concussion are non-specific. As such, clinicians are better able to manage patients when a standardized clinical exam is performed to sub-type the driver(s) of symptoms. Aerobic exercise and multimodal rehabilitation have consistently shown to be a possibly effective means to manage this population; however, the optimal training prescription is unclear. Thus, there is a need to further examine the effectiveness of personalized rehabilitative treatments. Our primary aim is to evaluate the response to personalized therapy on recovery, as measured by The Rivermead Post-concussion Symptoms Questionnaire (RPQ) when compared to an active control.

**Methods:**

We will conduct a multi-center 12-week case-crossover randomized controlled trial. 50 participants will be recruited from out-patient University Health Network clinics and community-based clinical practices around the greater Toronto area. Participants will be randomized at baseline to Group A: a personalized care program followed by an active control or Group B: an active control followed by a personalized care program. Participants will be included should they be 21 years of age and older and have symptoms that have persisted beyond 4 weeks but less than 1 year. Participants will undergo 6-weeks of care in their respective streams. After 6-weeks, participants will undergo a re-examination. They will then crossover and undertake the alternative treatment for 6 weeks. At the end of 12 weeks, participants will undertake the endpoint examinations. The primary outcome will be the Rivermead Postconcussion Questionnaire (RPQ). The secondary outcomes will be changes in standardized clinical examination, Neck Disability Index (NDI), Patient Health Questionnaire (PHQ-9) and an electroencephalography (EEG) via NeuroCatch^™^. The statistical analysis to be performed is composed of an adjusted model using an analysis of variance, specifically using an unpaired t-test to test for associations between variables and outcomes.

**Discussion:**

Given the recommendations from reviews on the topic of rehabilitation for adults with persistent concussion symptoms, we are undertaking a controlled trial. The documented high costs for patients seeking care for persistent symptoms necessitate the need to evaluate the effectiveness of a personalized rehabilitative program compared to the current standard of care.

**Trial registration:**

ClinicalTrials.gov ID: NCT06069700.

**Supplementary Information:**

The online version contains supplementary material available at 10.1186/s12883-024-03700-5.

## Background

Disabilities secondary to traumatic brain injury (TBI) are a major source of burden. Although the majority of patients who suffer a mild TBI (mTBI) recover, timelines are highly variable [1]. A recent large working group identified that up to 82% of patients report at least one symptom 6–12 months after a mTBI and that headaches appear to be the most common and pervasive symptom endorsed [2, 3]. Additionally, Graff et al. (2019) noted that mTBI has a long-term impact on labour markets. They found that 43% of patients don’t return to ordinary work 5-years post-injury [4]. The implications for this are significant for the person, the healthcare system and society. Thus, it’s imperative we further evaluate potentially beneficial treatments.

Unfortunately, there remains no agreed-upon biological basis for the symptom clusters reported by patients. The aggregate of symptoms reported by those suffering persistent symptoms is viewed as being non-specific. Leddy et al. (2015) showed that symptoms reported on a 22-item Post-concussion Symptom Scale questionnaire following a mild traumatic brain injury could not reliably discriminate between concussion subtypes [5]. Symptoms reported after a head injury traditionally have been ascribed to the brain, particularly cognitive symptoms. However, even cognitive complaints, including issues with concentration, brain fog and memory deficits have been reported after whiplash injuries [6]. This strongly suggests the need for clinicians managing this population to have performed a thorough examination.

Leddy et al. (2021) proposed a systematic clinical examination to help identify one or more clinical profiles of the post-concussion patient [7]. The identified clinical profile(s) would theoretically assist the treatment provider(s) in providing targeted therapies to optimize recovery. Leddy et al. (2021) noted that specifically including graded physical exertion testing will assist with identifying patients who exhibit cardinal features of an autonomic dysfunction concussion profile when they demonstrate exercise intolerance. Exercise intolerance is defined as an inability to exercise at an age-appropriate threshold due to the exacerbation of concussion symptoms [7]. When patients can exert themselves and achieve near their age-appropriate maximum heart rate without exacerbation of symptoms, then the etiology or justification for persisting concussion symptoms is thought to be due to an alternative problem such as a cervical spine disorder, vestibular-ocular impairment, or mood/cognition related issues [7].

Numerous pharmacological and non-pharmacological treatments have been utilized and studied in an attempt to treat persistent concussion symptoms. Specifically, symptom-limited aerobic exercise appears to be a staple therapy included in most trials assessing improvements in persistent symptoms [8, 9]. Further, the most recent consensus statement on concussion in sport from the 6th International Conference on Concussion in Sport recommends patients be treated for persisting symptoms via symptom-limited aerobic exercise [10]. Rytter et al. (2021) noted that following a systematic review and meta-analysis of 19 randomized clinical trials, there is weak evidence for nonpharmacological interventions, including advice, use of graded physical exercise, manual therapy to the neck, vestibular therapy and interdisciplinary rehabilitation [11]. Continuing, Moser et al. (2023) reported that multimodal rehabilitative therapy consistently demonstrates the strongest level of evidence among the various nonpharmacological treatments studied to date for physically dominant persistent symptoms [12]. Multimodal therapy was defined as the use of multiple treatment modalities concurrently for patients provided by either an interdisciplinary team or a single practitioner [12]. Similarly to the conclusions of Rytter et al., the authors note that the majority of trials suffer methodological weakness such as consistently failing to include a treatment control group, not recruiting sufficient participants to properly power the trial, or failing to use reliable and validated clinical outcome measures [12]. Given the preliminary findings for exercise and multimodal therapy, they suggest further research in this area.

### Objectives

The present study was developed to better understand the effects of personalizing rehabilitative treatments for patients with persistent concussion symptoms. Our primary aim is to evaluate the effectiveness of a personalized rehabilitation program based on subgroup classification, as measured by The Rivermead Post-concussion Symptoms Questionnaire (RPQ) when compared to an active control. Specifically, the objective is to assess whether personalizing rehabilitation following a standardized examination affords improved recovery when compared to an active control, which is reflective of the current standard of care for this population. Given symptoms are non-specific, we hypothesize that participants undergoing a personalized rehabilitation program derived from subgroup classification following a standardized exam will demonstrate clinically meaningful and statistically significant improved Rivermead Postconcussion Questionnaire scores when compared to the active control whose therapy approach is based on general rehabilitation recommendations.

Our secondary aim is to examine participants’ cognition Pre and Post-treatment (6 and 12-week follow-up points) via a quantitative electroencephalogram (EEG). Due to the various ostensible drivers of impaired cognition in this population, such as neck pain, sleep impairment, and altered mood, the EEG will serve as an objective cognitive evaluation.

Our tertiary aim is to examine at baseline and the follow-up points, participants’ Neck Disability Index, Patient Health Questionnaire (PHQ-9) and electrophysiological brain activity changes as measured by quantitative EEG. These outcomes will be compared to the standardized clinical examination findings as well as the RPQ. This will be done to identify any possible neuro-biomarker (abnormal ERPs), level of perceived neck disability or baseline level of depression that is predictive of treatment responders versus non-responders.

## Methods

### Ethics approval

Ethics approval for this clinical trial has been granted by the University Health Network Research Ethics Board (#22-5560). All participants are required to provide both verbal and written informed consent before commencing any experimental procedures. [protocol version 4, January 13, 2023]

### Study design

We will be conducting a 12-week case-crossover randomized controlled trial (see Table 1). At baseline, following the standardized examination, completion of the primary outcome measure (RPQ), baseline quantitative EEG and secondary outcome measures (NDI and PHQ-9), participants will be randomized to either Group A: a personalized care program followed by an active control or Group B: an active control followed by a personalized care program. No washout period will be used between interventions. It was assumed that given both interventional groups have a focus on rehabilitative therapy that no wash-out period was needed. Participants will undergo 6 weeks of care in their respective streams. After 6 weeks, participants will undergo a re-examination. They will then crossover and undertake the alternative treatment for 6 weeks. At the end of 12 weeks, participants will undertake the endpoint examinations.

**Table 1:** Enrolment schedule, interventions and assessments

	Study Period						
	Enrolment/Baseline assessment	Allocation	T1	Mid-point assessment (6 weeks)	Cross-over	T2	End-point assessment (12 weeks)
**TIMEPOINT**							
**Eligibility**	X	X					
**Informed consent**	X						
**INTERVENTIONS**							
**Personalized care program**			X	X	X	X	X
**Usual care program**			X	X	X	X	X
**ASSESSMENTS**							
**RPQ-3**	X			X			X
**RPQ-13**	X			X			X
**NDI**	X			X			X
**PHQ-9**	X			X			X
**EEG**	X			X			X

T1: \First 6 weeks of rehabilitation; T2: Second 6 weeks of rehabilitation; RPQ: Rivermead Postconcussion Questionnaire; NDI: Neck Disability Index; PHQ-9: Patient Health Questionnaire; EEG: Electroencephalography via NeuroCatch platform

### Population

Participants will be recruited from external community concussion clinics around the greater Toronto area as well as by internal advertisement at the University Health Network. Internal advertisements will consist of posters posted at the University Health Network. Recruitment via external community clinics will be a result of referrals by physicians working with patients who are suffering persistent concussion symptoms. Participants will undergo therapy at either the KITE Innovations and Rehabilitation Clinics located within the Toronto Rehabilitation Institute, Toronto, Ontario, Canada or the Pain and Wellness Centre, an interdisciplinary pain management clinic located just north of the city of Toronto, Ontario, Canada.

### Inclusion criteria

- 21-years and older.
- Meet the definition of postconcussion syndrome as defined by Tator et al. (2016), which requires a participant to report any 3 symptoms or more (from an inclusive list of the 40 most commonly reported persisting symptoms) lasting at least 1-month following the diagnosis of a concussion [13]. Concussion was defined according to the 6th International Consensus Statement on Concussion in Sport [10].
- Have adequate language skills in English to read and take part in rehabilitation treatment programs.
- Demonstrate an objective impairment on the baseline standardized exam, placing the participant into one of the three subgroups (autonomic, cervical or vestibulo-ocular).

### Exclusion criteria

- In-patients at Toronto Rehabilitation Institute or any other affiliated University Health Network clinics.
- Participants will be excluded should their clinical examination be unremarkable for any positive physical findings and therefore their dominant symptomatology places them into the Affective / Cognition subgroup described by Leddy et al. [7].
- Have a chronic infectious disease.
- Uncontrolled hypertension.
- Other neurological disorders (not attributed to their primary diagnosis).
- Cancer treatment (other than basal cell carcinoma), craniotomy, or refractory subdural hematoma.
- long-term use of psychoactive medications that would compromise their ability to comprehend and perform study activities.
- Those with pacemakers or elevated cardiovascular risk.
- Ongoing litigation surrounding their injury.
- Have been diagnosed with a moderate or severe brain injury prior to enrollment, or their post-concussive symptoms have persisted beyond 12 months.

### Randomization and masking

We will use simple randomization to allocate participants to one of two sequences of interventions: Group A: personalized care program followed by active control; or Group B: active control followed by personalized care program. The study coordinator (NM) conducted the randomization using a randomized table generator (GraphPad Software Inc, La Jolla, CA). Randomization will be concealed; no other study personnel will be aware of the intervention assignments. However, it was not possible to blind the study coordinator (NM) as they are one of the main treating clinicians. Additionally, it was not possible to ensure adequate blinding of the participants, given the necessary thorough description of the treatment procedures that occurred during the informed consent process.

### Assessments / procedures

Participants will undergo a comprehensive clinical evaluation at the KITE Innovations and Rehabilitation Clinics or at the Pain and Wellness Centre to differentiate their post-concussive subgroup(s) [7]. Evaluation will consist of recommended elements of a standardized clinical physical examination outlined by Matuszak et al. (2016) [14]. Specifically, the study coordinator (NM) will perform all components of the examination including a neurological exam consisting of cranial nerve screen, motor testing of the upper and lower extremities and deep tendon reflexes; a musculoskeletal examination assessing for tenderness over the head and neck, range of motion of the cervical spine and Spurling test; joint position sense error test (JPSE) of the cervical spine in flexion, extension, lateral flexion and rotation, which has shown to be a reliable and relevant measure to distinguish normal and neck pain patients [15–17]; balance/coordination examination assessing static and dynamic balance via the Balance Error Scoring System (BESS), and tandem walking with eyes open and closed [18]. The BESS consists of three static stances performed on both a firm and foam surface. The BESS has been shown to have moderate to good reliability in assessing static balance as well as has been shown to detect balance deficits in participants with concussion [19]. Continuing, a vestibular-ocular examination consisting of evaluation of the eyes in eight positions, evaluating nystagmus, saccades, smooth pursuit and near point convergence/accommodation. Additional tests will be included if dizziness or imbalance are present and will include: orthostatic vital signs via supine-to-stand stress test; Dix-Hallpike maneuver and assessment of dynamic visual acuity [20].

Lastly, a physical exertion test will be performed via the Buffalo Concussion Treadmill Test (BCTT) protocol to specifically examine exercise intolerance. The BCTT is a well-established aerobic exercise test designed to assess exercise tolerance [21]. BCTT has shown clinical utility in identifying those likely to suffer persistent symptoms and the test is an excellent guide for exercise prescription following a concussion [22–24]. In addition to the baseline, 6-week and 12-week assessments, the BCTT will be performed weekly on participants in the personalized care stream who have been placed into the autonomic subgroup. This will be done to ensure proper advancement of their target heart rate participants are exerting themselves aerobically during training as rehabilitation and recovery progresses.

A positive or failed BCTT, placing the participant into the autonomic subgroup, will be defined as an inability to exercise at an age-appropriate heart rate threshold due to the exacerbation of concussion symptoms [15]. Exacerbation of symptoms will be defined as an increase of three points or more of their reported symptoms from baseline during exercise on an 11-point numerical rating scale (0–10). When patients can exert themselves and achieve near their age-appropriate maximum heart rate without exacerbation of symptoms, then the etiology or justification for persisting concussion symptoms will be ascribed to alternative problems [7].

A positive cervical spine screen, placing the participant into the cervical subgroup, will be defined as having an abnormal cervical spine range of motion with palpatory findings of facet joint restrictions and pain with supine facet joint motion palpation, cervical myofascial trigger points, or abnormal joint position error testing [14].

Finally, a positive vestibular or oculomotor screen, placing the participant into the vestibulo-ocular subgroup, will be defined as an abnormal vestibulo-oculomotor screen (VOMS) or abnormal static and dynamic balance testing as assessed via the Balance Error Scoring System and tandem walking, respectively [14].

As an additional element, participants’ cognition will be specifically examined through a rapid advanced quantitative electroencephalogram (EEG) via the NeuroCatch™ brain vital sign framework. Conventional EEG and event-related potentials (ERP) methods and analysis are time-consuming and typically restricted to controlled laboratory settings. This makes its use to help objectively guide a clinician’s management decision inaccessible to routine clinical use. Hajra et al. (2016) have detailed the framework of the brain vital sign in young and older individuals [25]. The framework translated well-established ERP responses into a portable, rapid, automated and easy-to-use evaluation method. It also incorporates a normative existing comparison framework analogous to existing vital sign metrics, such as that for blood pressure. Quantitative EEGs have repeatedly shown that concussions cause objective measurable changes in brain activity and therefore the functions of the structures from which they originate [26]. Specifically, Fickling et al., (2019) showed abnormal ERPs in elite male junior hockey players acutely following a concussion [27]. They also observed persistent impairments in ERP, specifically P300, at the time of return to play. Fickling et al. (2019) also found that players who did not sustain a concussion during the season still demonstrated abnormal ERPs (N400) at the end of the season, representing possible cognitive impairment as a result of cumulative subconcussive impacts [27]. Additionally, Ozen et al. (2013) demonstrated, in young adults who sustained a concussion a minimum 1-year before testing, a negative correlation between abnormal ERP (decreased amplitude of P300) and lingering impairments in cognitive processing, memory, and concentration [28]. NeuroCatch™ is a portable quantitative EEG and will be performed at the KITE Innovations and Rehabilitation Clinics. NeuroCatch™ acquires and reports on event-related potentials (ERPs) derived from electroencephalogram (EEG). As part of the brain vital sign framework, three ERPs (brain vital sign), auditory N100 (auditory sensation); auditory oddball P300 (basic attention); and auditory speech processing N400 (cognitive processing) will be evaluated.

### Intervention

The study coordinator (NM) will perform all components of the listed assessments/procedures. The interventions will be delivered by advanced-trained chiropractors at the Pain and Wellness Centre and the study coordinator (NM) at the KITE Innovations and Rehabilitation Clinics.

### Personalized care program

On the first therapy session, participants in the personalized care stream will be provided reassurance and psychoeducation via the bio-psycho-social model on understanding persistent symptoms consistent with usual care. Additionally, they will be afforded up to 12 treatments over the course of 6 weeks. The time frame of 6 weeks was selected given the treatment structure of prior clinical trials examining the effectiveness of physical interventions to address physical symptoms such as headaches, dizziness and neck pain [29]. Treatments will be standardized; however, given the heterogeneity of symptoms, components of the treatments will be personalized to the participants based on what subgroup they’re classified into at baseline. Personalized rehabilitative care differentiated by subgroups is outlined below:

- **Autonomic group -** Participants will receive 60 min twice per week of supervised progressive sub-symptom aerobic exercise, as well as mindfulness-based training. As previously indicated, the target heart rate will be determined by the BCTT. This test will be done weekly to ensure the timely advancement of the target heart rate while participants’ rehabilitation and recovery progress. Participants will be instructed on various mindfulness-based techniques they can perform at home; however, in the clinic twice per week, a box-breathing technique will be utilized. Participants will be instructed to perform a minimum of 20 min of sub-symptom aerobic exercise as well as 20 min of mindfulness-based training daily outside the supervised exercise sessions.
- **Cervical group -** Participants will receive bi-weekly physical therapy to the cervical spine. Physical therapy will include soft tissue therapy directed to the cervical myofascial tissues and graded cervical spine facet mobilizations. Participants will also receive supervised 20 min of progressive neck isotonic strengthening exercises. Additionally, participants will be instructed to also perform daily 20 min of general neck stretches, range of motion exercises and neck strengthening exercises outside the supervised exercise sessions.
- **Vestibulo-Ocular group -** Patients will receive bi-weekly individualized oculomotor, vestibular and balance exercises including adaptation exercises, gaze stability exercises, visual-vestibular integration exercises, habituation exercises, and static and dynamic balance exercises. They will also be instructed to perform daily 20 min of individualized vestibular and oculomotor exercises based on their clinical exam outside the supervised exercise sessions.

In cases where participants are classified into more than one subgroup, they will receive the listed treatments provided to the sub-groups they are classified under. However, the frequency (twice per week) and duration (6-weeks) of care will not change regardless of subgroup determination.

Lastly, participants will be asked to refrain from seeking additional therapy; however, should they choose to undertake additional therapy outside of the trial they will be asked to document the modality and frequency of said therapy in the provided treatment adherence calendar that every participant receives at the start of the trial.

### Active control (usual care program)

All participants in the active control group will receive ‘usual care’, (one-hour session once a week for 6 weeks). The usual care model will consist of a single-hour session, provided at the first visit following the baseline assessment, devoted to reassurance and psychoeducation via the bio-psycho-social model on understanding persistent symptoms, including advice on adaptive illness behaviours, such as gradually resuming premorbid activities and avoiding excessive rest and “all-or-nothing behaviour”. Continuing, they will be educated on the fact that symptoms can be exacerbated not only with physical activity but also with extended cognitive load, which occurs commonly during working hours and social situations. As such they will be informed to take 5-10-minute breaks every hour if working and to find quiet spaces during busy social situations periodically. Additionally, as part of their weekly one-hour therapy sessions, participants will be afforded 30 min supervised symptom-limited aerobic exercise sessions. They will also be instructed to perform 20 min of symptom-limited aerobic exercise daily outside the supervised exercise sessions. Symptom-limited exercise will be defined as any form of aerobic exercise the participants will be able to perform without causing a greater than 2-point change in total symptoms as rated on an 11-point pain numerical rating scale (0–10) during their training. They will be instructed to rate their symptoms at rest before the exercise period and then exercise aerobically for 20 min without worsening their symptoms. They will be instructed to stop exercising and rest should their baseline resting symptoms exceed a 2-point increase during the exercise session. Additionally, when clinically indicated based on the standardized exam, participants will receive 20 min of general neck active range of motion exercises and stretching along with 20 min of generalized static and balance exercises. Participants will be advised to perform the neck and balance exercises two to three times per week outside of the supervised exercise sessions.

### Outcomes

#### Primary outcome

Participants’ overall symptomatology will be evaluated with the Rivermead Post-Concussion Symptoms Questionnaire (RPQ). RPQ is a simple questionnaire composed of 16 concussion symptoms rated by patients which evaluates cognitive, physical and emotional symptoms according to their severity. It has been demonstrated that when the entire 16-item questionnaire is summated together there is a poor overall fit to the Rasch model, suggesting all 16 items do not tap into the same underlying construct [30]. However, when split into two separate scales, the RPQ-3 has shown moderate test-retest reliability and RPQ-13 has shown good test-retest reliability and each set of items forms a unidimensional construct for people with head injury at three months post-injury [30, 31]. The RPQ-3 is associated with early symptom clusters of post-concussive symptoms (scored 0–12), and the RPQ-13 is associated with having a greater impact on participation, psychosocial functioning, and lifestyle (scored 0–52), with a higher score representing a greater impact [31].

#### Secondary outcomes

The secondary outcome measures include a quantitative EEG measuring three ERPs (brain vital sign framework), the Neck Disability Index (NDI), and the Patient Health Questionnaire-9 (PHQ-9).

### Brain vital sign

Brain vital sign will be measured by rapid advanced electroencephalography (EEG) via NeuroCatch™. Specifically, the three ERPs, auditory N100 (auditory sensation); auditory oddball P300 (basic attention); and auditory speech processing N400 (cognitive processing) will be evaluated. Standardized normative data exists for all three ERP responses [32–34].

### Neck Disability Index (NDI)

Neck-related disability will be measured via the NDI. The NDI is a 10-item questionnaire, which examines the impact of self-reported neck pain on various activities of daily living. The responses are summed for a total ranging from 0 to 50 with a higher score representing a more severe perceived disability. The questionnaire has demonstrated reliability, construct validity and responsiveness to change in various populations [35].

### Patient Health Questionnaire-9 (PHQ-9)

A screening form for depression and anxiety via the PHQ-9 will be administered. The PHQ-9 has been shown to have adequate reliability and good validity for the concussion population as well as the spinal pain population [36–39].

All outcome measures will be compared to participants’ values at baseline, 6-week re-assessment and trial end-point assessment.

### Statistical analysis

The primary outcome is a change in symptoms following rehabilitative interventions using the RPQ. Previous research examining the internal construct validity of the Rivermead Post-Concussion Symptom Questionnaire suggested that a score of 2 or more on at least 3 of the 16 symptoms represents an unfavourable outcome [40]. We chose to calculate the study sample size by assuming an effect size. Unfortunately, to date, no validated change scores exist, thus we prescribed a cut-off of 15% improvement as minimal clinically importance difference (MCID). This translates into a change of 2 points on the RPQ-3 and a change of 8 points on the RPQ-13. This agrees with prior clinical literature examining changes post-intervention on concussion symptoms [41]. Based on this effect size, a power of 80%, a significance level at *p* < 0.05 and a two-sided t-test, we calculated that a sample size of 50 participants was necessary [42].

Descriptive statistics will be applied to all outcome measures to assist in summarizing the results. Participant demographic data will be compared to assess compatibility between groups, specifically before the crossover point.

The statistical analysis conducted in this research is composed of an adjusted model using an analysis of variance to test for associations between variables and outcomes (two groups [personalized care or usual care] x 3 times [baseline, week 6 and 12]) [42]. The statistical analysis conducted in this study will be made using SAS OnDemand for Academics 2021. The data set is composed of six variables, two independent variables –personalized care group or usual care group – and four dependent variables – age, sex, and result of treatment on the RPQ graded as total score and sub-classification (RPQ-3 and RPQ-13). A 2-way analysis of covariance (ANCOVA) (two groups [personalized care or usual care] x 3 times [baseline, week 6 and 12] x baseline secondary outcomes [ERPs, NDI and PHQ-9] will be used to examine the effects of the rehabilitation programs while controlling for these covariates. Lastly, an unpaired t-test will be used to assess the effects of the rehabilitation programs on the secondary outcomes. In addition to the per-protocol analysis, an intention-to-treat analysis will be conducted.

All unintended effects, harms and/or dropouts during the program will be recorded.

## Discussion

The results of the project may have implications for helping to establish real-world personalized treatment protocols for the rehabilitation of persistent concussive symptoms. Currently, single-therapy trials make up the vast majority of studies examining the efficacy of treatments on recovery from persistent concussion symptoms. Additionally, to date, the few trials examining the effects of sub-symptom aerobic exercise have only been compared to a full-body stretching program [43, 44]. Given the consistently positive responses that exercise seemingly affords this population [8, 9], it is imperative to continue to evaluate optimal parameters. This is especially important because of the well-documented high degree of so-called stubborn exercise responders. The literature has consistently shown that should aerobic training volume and or intensity not be sufficiently intense for the individual, then no observable adaptions will occur [45]. In the present trial, both treatment programs afford participants active rehabilitation, with a focus on aerobic exercise training.

We hope to demonstrate that a personalized rehabilitative treatment protocol, which targets specific symptoms from the global concussion umbrella will lead to superior outcomes. Additionally, we hope that through the undertaking of the trial, we identify potential biomarkers, as measured by EEG and the standardized physical assessment, that can be predictive of response to therapy. Finally, we hope to positively contribute to the body of knowledge supporting therapies to treat impairments in post-concussive patients, which have been labelled as permanent.

### Electronic supplementary material

Below is the link to the electronic supplementary material.

Supplementary Material 1

## Data Availability

No datasets were generated or analysed during the current study.

## Abbreviations

RPQ: Rivermead Postconcussion questionnaire
EEG: Electroencephalography
ERP: Event-related potential
MCID: Minimum clinically important difference
NDI: Neck disability index
PHQ-9: Patient health questionnaire
TBI: Traumatic brain injury
mTBI: Mild traumatic brain injury
JPSE: Joint position sense error test
BESS: Balance Error Scoring System
BCTT: Buffalo Concussion Treadmill Test
